# DNA methylation of DKK‐1 may correlate with pathological bone formation in ankylosing spondylitis

**DOI:** 10.1002/iid3.911

**Published:** 2023-07-07

**Authors:** Yu‐Cong Zou, Zhi‐Jun Wang, Li‐Cheng Shao, Zhi‐Hong Xia, Yi‐Feng Lan, Zhi‐Hui Yu, Jia‐Yu Yao, Zi‐Rui Luo

**Affiliations:** ^1^ Department of Rehabilitation The 5th People's Hospital of Foshan City Foshan Guangdong Province China; ^2^ Deaprtment of Rehabilitation The 5th Affiliated Hospital of Foshan University Foshan Guangdong Province China; ^3^ Department of Internal Medicine The 5th People's Hospital of Foshan City Foshan Guangdong Province China; ^4^ Department of Radiology The 5th People's Hospital of Foshan City Foshan Guangdong Province China; ^5^ Department of Laboratory medicine The 5th People's Hospital of Foshan City Foshan Guangdong Province China

**Keywords:** ankylosing spondylitis, disease severity, DKK‐1, DNA methylation, pathological bone formation

## Abstract

**Objective:**

To investigate DNA methylation (DNAm) status of dickkopf‐associated protein 1 (DKK‐1) in ossified hip capsule synovium and serum among patients with ankylosing spondylitis (AS).

**Methods:**

Western blot was applied to detect the level of DKK‐1 protein expression in hip joint capsule tissues from four patients with AS as well as four patients with femoral neck fracture (FNF) caused by trauma as control. DKK‐1 gene promoter methylation (GPM) was examined by methylation‐specific polymerase chain reaction. Reverse transcription‐polymerase chain reaction was performed to examine the messenger RNA (mRNA) levels of DKK‐1, β‐catenin, and Wnt3a in both tissue and serum. The DNAm status of serum DKK‐1 was measured among 36 patients with AS and syndesmophytes (AS + syndesmophytes group), 40 patients with AS but no syndesmophyte (AS group), and 42 healthy individuals (control group). Also, the serum levels of DKK‐1 were measured by enzyme‐linked immunosorbent assay. The modified New York criteria (mNYC) together with the modified Stoke Ankylosing Spondylitis Spinal Score (mSASSS) were adopted to examine the radiographic progression of AS. The receiver operating characteristic (ROC) curve was applied to investigate the diagnostic value of the methylation rate of DKK‐1 with regard to radiographic progression.

**Results:**

The expressions of DKK‐1 protein and mRNA in hip joint capsule tissues of AS patients were significantly lower, while DKK‐1 GPM rate, β‐catenin mRNA, and Wnt3a mRNA were markedly higher when compared with FNF group. For serum samples, the DKK‐1 methylation rate was significantly higher in AS+ syndesmophytes group in contrast to AS group and healthy controls. Serum levels of DKK‐1 protein and mRNA in AS with syndesmophytes group were markedly decreased, while β‐catenin mRNA and Wnt3a mRNA expressions were significantly increased than AS with no syndesmophyte group and the healthy control group. AS patients in Grade 4 showed a significantly higher serum DKK‐1 GPM rate than those in Grade 3 based on mNYC. Serum DKK‐1 GPM level was markedly and positively correlated with mSASSS. Serum levels of DKK‐1 in AS+ syndesmophytes group were markedly lower compared with AS but no syndesmophyte group and healthy controls. ROC curve analysis indicated that serum DKK‐1 methylation rate serves as a decent indicator for AS radiographic progression.

**Conclusion:**

DNAm of DKK‐1 may correlate with pathological bone formation in AS, which may provide new strategies for the treatment of AS abnormal bone formation.

## Graphical abstract

1. Serum dickkopf‐associated protein 1 (DKK‐1) gene promoter methylation (GPM) level was markedly and positively correlated with modified Stoke Ankylosing Spondylitis Spinal Score. 2. DNA methylation of DKK‐1 may correlate with pathological bone formation in ankylosing spondylitis (AS), which may provide new strategies for the treatment of AS abnormal bone formation.

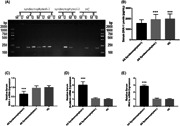

**Abbreviations**
AS: ankylosing spondylitis
DKK‐1: dickkopf‐associated protein 1

## 1 INTRODUCTION

Being a chronic inflammatory disease, ankylosing spondylitis (AS), mainly involves the axial joint with unclear pathogenesis, with the characteristics of pathological ossification leading to spine or joint stiffness and permanent disability.^1^ Inflammatory back pain, spinal fusion as well as immobilization due to entheseal pathological new bone formation are the most distinctive manifestations of AS.^2^ Given that the majority of AS patients are young/middle‐aged men, their disability would be a heavy burden to the patients' family and even society, leading to large socioeconomic costs.^3^ Recently, incremental studies/medications have paid attention to the inhibition of inflammation along with the relieving of pain. However, there is still a lack of treatments targeting pathological bone formation and satisfactory prognosis of axial structural damage.^4^ In addition, the pathogenesis of entheseal new bone formation that finally causes bony bridging has remained unclear.

As one of the most studied proteins in AS, dickkopf‐associated protein 1 (DKK‐1) can inhibit Wnt/β‐catenin pathway, acting as a pivotal regulator of bone mass, the expression level of which is associated with osteopenia/high bone mass.^5^ Numerous clinical and experimental studies have shown that abnormal formation of osteophytes is associated with the decreased DKK‐1 expression or dysfunction of DKK‐1 in AS. Various studies have demonstrated that serum DKK‐1 levels was significantly lower in AS patients compared with the control group.^6^, ^7^, ^8^ DKK‐1 has been found to be dysfunctional in AS patients in comparison with rheumatoid arthritis (RA) patients and healthy control group.^9^ In our previous studies, we also found that functional dickkopf‐1 concentrations are negatively related to radiographic severity of AS.^10^ We also found silencing DKK‐1 enhances the proliferative/osteogenic potential of fibroblasts that were isolated from AS.^11^ In TNFtg mice model, DKK‐1 blockade promoted collagen type X expression, hypertrophic chondrocyte formation as well as sacroiliac joint ankylosis.^12^ However, the mechanism that why DKK‐1 is downregulated and dysfunctional in AS remains unclear.

Epigenetic has been recently regarded as one important gene regulatory mechanism that controls the accessibility of chromatin to transcriptional regulatory factors, thereby tuning gene expression without changing the underlying DNA sequence.^13^ Epigenetic events can be influenced by the environment, are dynamic but also heritable and are in the end responsible for significant variation between cells and tissues in one organism, independent of the identical genotype between all diploid cells.^14^ Epigenetics studies the heritable expression of genes from many aspects^15^ including DNA methylation (DNAm), noncoding RNA, histone posttranslational modification together with chromatin remodeling, and so forth.^16^ As one of the most common forms of epigenetic modification, DNAm is involved in gene expression and mediated by DNA methyltransferases (DNMTs), and an imbalance in genomic methylation.^17^ The gene promoter region is rich in the main region of DNAm CpG islands, which are located in genetic regulatory elements and are usually defined as regions with a length greater than 200 base pairs and a G + C content greater than 50%.^18^ DNAm starts at one end of the islands and continues to gene promoters and initiation sites, altering the three‐dimensional configuration of the DNA and inhibiting its interaction with transcription factors, ultimately silencing gene expression. In contrast, hypomethylation promotes gene expression.^18^

Recent studies have proved that DNAm exerts a significant effect on the occurrence/development of AS.^19^, ^20^, ^21^, ^22^ Besides, recent studies have also shown that DKK‐1 methylation can promote the progression of cervical cancer,^23^ rectal cancer,^24^ leukemia,^25^ and other diseases. However, there are no studies available illustrating the potential relationship between DKK‐1 methylation and AS abnormal new bone formation.

Therefore, our study was aiming to detect DKK‐1 protein expression along with its DNAm status in ossified hip joint capsule as well as the serum of AS patients to provide a basis for the mechanism of pathological osteogenesis and treatment of AS.

## 2 MATERIALS AND METHODS

### 2.1 Tissue sample and serum sample collection

For tissue sample collection, four AS patients with hip ankylosis in our hospital undergoing hip replacement, hip arthroplasty or lesion excision were selected. For the control group, four patients with femoral neck fractures (FNFs) due to trauma were selected to undergo hip replacement as control (Tables 1 and 2). For serum sample collection, we also enrolled 36 patients with AS and syndesmophytes (AS + syndesmophytes group), 40 patients with AS but no syndesmophytes (AS group), as well as 42 healthy individuals (control group). All AS patients met the modified New York criteria (mNYC) in 1984.^26^ The basic demographic data of the three groups are listed in Table 3. The presence of syndesmophyte existence in AS patients was confirmed by two experienced radiologists. Informed consent was obtained from all the patients. The 5th People's Hospital of Foshan City ethics committee already granted approval (Ethical Approval Number: 20220019).

**Table 1:** Basic clinical data for AS patients for collecting tissue samples.

No.	1	2	3	4
Age (years)	40	35	39	38
Sex (male/female)	Male	Male	Male	Male
BMI (kg/cm^2^)	20.4	21.2	20.5	21.6
Duration of disease (years)	6.9	7.3	7.0	6.4
BASDAI	4.0	3.9	3.9	4.2
NSAIDs used	Yes	Yes	Yes	Yes
DMARDs used	MTX + SSZ	MTX + SSZ	MTX + SSZ	MTX + SSZ
Corticosteroids used	No	No	No	No
TNF‐blockade used	No	No	No	No

Abbreviation: BASDAI, bath ankylosing spondylitis disease activity index; BMI, body mass index; DMARD, disease modifying antirheumatic drug; MTX, methotrexate; NSAID, nonsteroidal antiinflammatory drug; SSZ, sulfasalazine; TNF, tumor necrosis factor.

**Table 2:** Basic clinical data for FNF patients (control) for collecting tissue samples.

No.	1	2	3	4
Age (years)	42	39	35	40
Sex (male/female)	Male	Males	Male	Male
BMI (kg/cm^2^)	22.7	21.5	20.8	22.3

Abbreviation: BMI, body mass index.

**Table 3:** Clinical and demographic characteristics of AS patients (serum samples).

	AS with syndesmophytes (*n* = 36)	AS without syndesmophytes (*n* = 40)	Healthy control (*n* = 42)	*p* Value
Age (years)	36.1 ± 7.5	35.8 ± 6.9	37.0 ± 7.0	.618
Sex (female/male)	7/29	10/30	10/32	.834
BMI (kg/m^2^)	21.6 ± 3.2	22.0 ± 2.8	21.8 ± 2.9	.524
Time since diagnosis –years (mean [SD])	9.0 ± 6.6	8.8 ± 5.9	/	.177
Drugs—*n* (%)				
NSAID continuous	15 (42%)	25 (63%)	/	.332
MTX	12 (33%)	14 (35%)	/	.999
SSZ	19 (54%)	25 (63%)	/	.706
TNFi	23(58%)	30 (68%)	/	.721

Abbreviation: BMI, body mass index; MTX, methotrexate; NSAID, nonsteroidal antiinflammatory drug; SSZ, sulfasalazine; TNFi, tumor necrosis factor inhibitor.

### 2.2 DNA extraction and sodium bisulfite modification

For tissue samples, the synovial tissues of the hip joint capsule were removed during aseptic surgery, stored in a heat preservation bucket containing an ice bag, immediately transported to the laboratory, and preserved in liquid nitrogen. The total DNA of hip capsule synovium in AS and FNF group was extracted using DNA extraction kit following the instructions of the manufacturer (Meiji Biotechnology Co., Ltd.). DNA extraction was performed for each serum sample (200 μL) using Serum/Plasma Circulating DNA Kit (TIANGEN). Subsequently, the eluted DNA (100 μL) was kept at the temperature of −20°C, preparing for the following experiments. Complying with the operating manual, the modification of extracted DNA was carried out using EZ DNAm‐Gold Kit (ZYMO Research Co) and stored at −20°C.

### 2.3 Detection of DKK‐1 gene promoter methylation (GPM)

The DKK‐1 GPM was detected using the method of methylation‐specific polymerase chain reaction (MSP). DNA modification was strictly carried out according to the instructions of DNAm transformation and purification kit (Kang Wei Century Biotechnology Co., Ltd.). Methylated primers were designed using MethPrimer 2.0 PrimerDesign. Table 4 shows the sequence of the primer. The reaction system of polymerase chain reaction (PCR) (20 µL) was composed of PCR master mix (10 µL), upstream (0.5 µL) and downstream (0.5 µL) primer, the treated DNA template (1.0 µL) as well as distilled water (8.0 µL). The circulation condition included 40 cycles of predenaturation (95°C, 5 min), denaturation (95°C, 15 s), annealing (60°C, 20 s), and extension (72°C, 30 s). The agarose gel (2%) was prepared for the electrophoresis (120 V, 30 min), and the results of electrophoresis were observed based on the gel imaging system. Methylation rate (%) = number of transformed DNA copies/(number of untransformed DNA copies + number of transformed DNA copies) × 100%.

**Table 4:** DKK‐1 primer sequence and fragment length.

Primer name	Primer sequence (5′ → 3′)	Fragment length (bp)
M primer‐F M primer‐R	TAGTAGTTGCGGAAGAGTCGC AAAAAACCAAAACTTATAAAACGCA	128
U primer‐F U primer‐R	GGGTAGTAGTTGTGGAAGAGTTGT AAAAAACCAAAACTTATAAAACAC	131

Abbreviations: M, methylated; U, unmethylated.

### 2.4 RNA isolation and real‐time PCR

Total RNA was extracted from hip tissue and serum using a total RNA isolation kit (Invitrogen Life Technologies), according to the manufacturer's protocol. Concentrations of total RNA were determined with a spectrophotometer. The specific primers for human DKK‐1 were 5′‐TGGCTCTGGGCGCAGCGGGAGCTACC‐3′ (sense) and 5′‐CGGCAAGACAGACCTTCTCCACAGTAAC‐3′ (antisense). For β‐catenin, the primers were: 5′‐CCACAAGATTACAAGAAACGGC‐3′ (sense) and 5′‐TGGATAGTCAGCACCAGGGT‐3′ (antisense). For Wnt3a, the primers were: 5′‐CAGTGCCTCGGAGATGGTG‐3′ (sense) and 5′‐GGTTAGGTTCGCAGAAGTTGG‐3′ (antisense). The amplification was performed for 25 cycles (94°C for 30 s, 56°C for 30 s, and 72°C for 30 s). The glyceraldehyde‐3‐phosphate dehydrogenase (GAPDH) sense primer is 5′‐GTATGTCGTGGAGTCTACTG‐3′ and the antisense primer was 5′‐TACTCCTTGGAGGCCATGTA‐3′. The relative target gene messenger RNA (mRNA) level was normalized to the GAPDH mRNA level.

### 2.5 Western blot

After the quantification of proteins, protein samples underwent sodium dodecyl sulfate (SDS)–polyacrylamide gel electrophoresis (PAGE), and then immunoblotted onto a nitrocellulose membrane. Subsequently, the proteins were incubated with Anti‐DKK‐1 antibody (Abcam) and β‐ actin antibody (Abcam) overnight at 4°C, followed by incubation with Goat Anti‐Rabbit IgG (H + L) (southern biotech) at room temperature for 1 h. After enhanced chemiluminescence development, the strip results were analyzed using Image J 8.0 software.

### 2.6 Enzyme‐linked immunosorbent assay (ELISA)

Venous blood samples collected from all participants were centrifuged and stored immediately at −80°C until analysis. DKK‐1 levels were assessed by commercial sandwich ELISA (R&D Systems Inc.). Intra‐ and inter‐assay coefficients of variation (CVs) were 2%–3% and 5%–9%, respectively.

### 2.7 Assessment of radiographic progression

Based on the mNYC^27^ and the modified Stoke Ankylosing Spondylitis Spinal Score (mSASSS),^28^ the radiographic progression for sacroiliac joint and spinal spondyloarthritis were correspondingly assessed. The grading system based on mNYC included: (1) Grade 0—normal; (2) Grade 1—suspicious changes; (3) Grade 2—minimal abnormality (small localized areas with erosion/sclerosis, without alteration in the joint width); (4) Grade 3—unequivocal abnormality (moderate/advanced sacroiliitis with ≥1 erosion, evidence of sclerosis, widening, narrowing/partial ankylosis); (5) Grade 4—severe abnormality (total ankylosis). Patients at Grades 2–5 in ≥1 sacroiliac joint were included. A higher grade of mNYC upon comparison between sacroiliac joints was used for measurement. The mSASSS, with the totals ranging from 0 to 72, was scored based on a lateral view of the anterior parts of lumbar & cervical spine, including (1) 1 point for squaring, erosion and/or sclerosis; (2) 2 points for syndesmophytes; (3) 3 points for bridging syndesmophytes.

### 2.8 Statistical analysis

GraphPad Prism 8.0 software was applied for data analysis. Student's *t* test was adopted for the comparison between groups. Besides, one‐way analysis of variance was used for the comparison among three groups. *χ* ^2^ test was conducted for the analysis of the categorical variables. Receiver operating characteristic (ROC) curve was adopted to assess the diagnostic value of the methylation rate of DKK‐1 among AS patients. The difference was considered statistically significant when *p* value was less than .05.

## 3 RESULTS

### 3.1 Comparison of DKK‐1 protein expressions and DKK‐1 GPM status among FNF and AS

**Figure 1:**
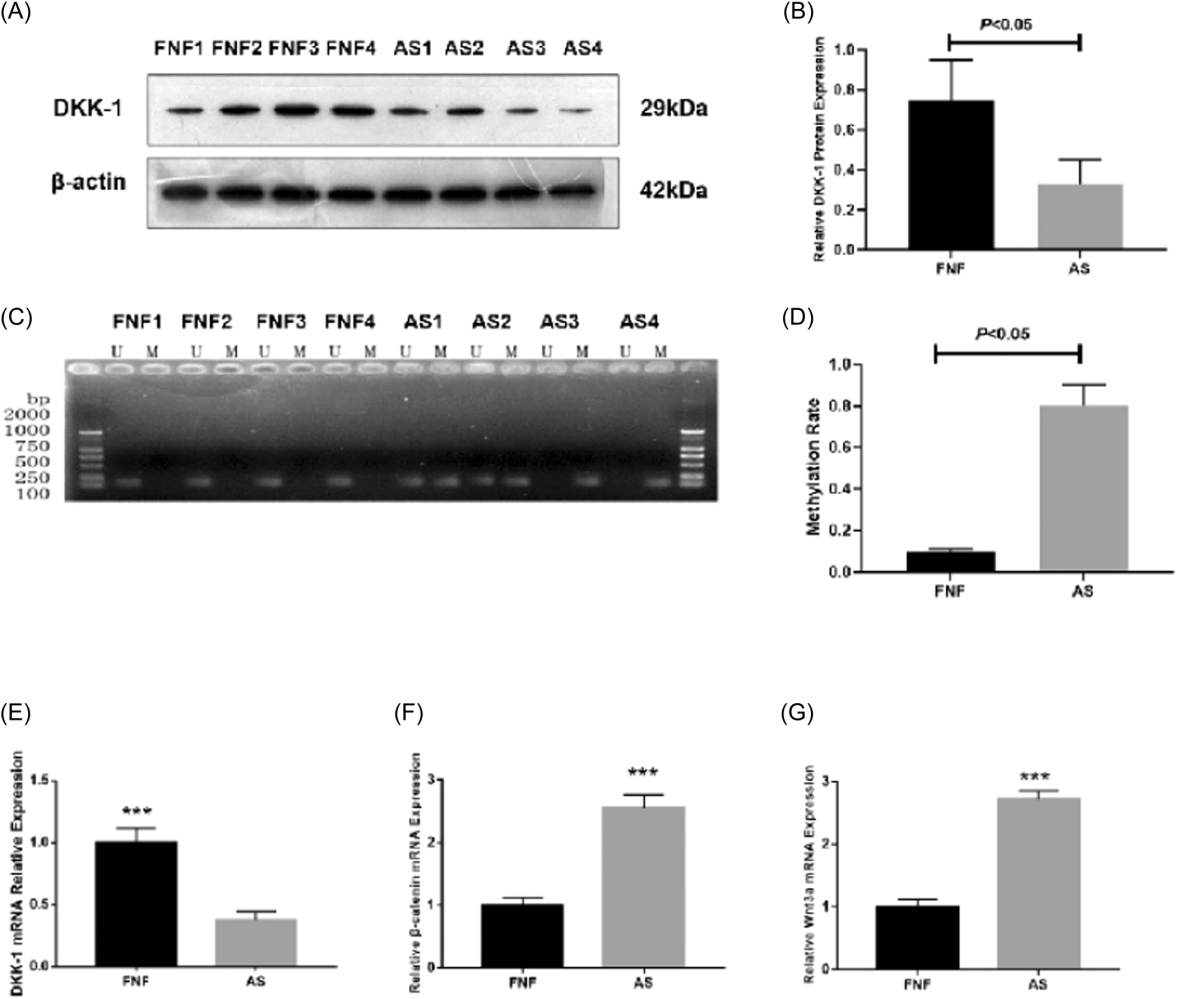
(A) Comparison of dickkopf‐associated protein 1 (DKK‐1) protein expressions between femoral neck fracture (FNF) (*n* = 4) and ankylosing spondylitis (AS) (*n* = 4) group. (B) Quantitative analysis of DKK‐1 protein expressions between FNF (*n* = 4) and AS (*n* = 4) group. (C) Methylation status of DKK‐1 gene promoter in tissue samples between FNF (*n* = 4) and AS (*n* = 4). (D) Comparison of methylation rate of DKK‐1 between FNF (*n* = 4) and AS (*n* = 4). (E) Comparison of DKK‐1 messenger RNA (mRNA) expressions between FNF (*n* = 4) and AS (*n* = 4). (F) Comparison of β‐catenin mRNA expressions between FNF (*n* = 4) and AS (*n* = 4). (G) Comparison of Wnt3a mRNA expressions between FNF (*n* = 4) and AS (*n* = 4). ***p < .001 versus FNF.

AS shown in Figure 1, compared with FNF group, the AS group demonstrated significantly lower DKK‐1 protein expressions (*p* < .05) (Figure 1A,B). Moreover, DKK‐1 gene promoters in FNF group were in nonmethylated state, while in the AS group, DKK‐1 gene promoters were partially methylated or fully methylated. There was a significant difference between the two groups (*p* < .05) (Figure 1C,D). The expressions of DKK‐1 mRNA in hip joint capsule tissues of AS patients were significantly lower (Figure 1E), while β‐catenin mRNA and Wnt3a mRNA expressions were markedly higher when compared with FNF group (Figure 1F,G).

### 3.2 The serum DKK‐1 protein and mRNA levels and GPM status

**Figure 2:**
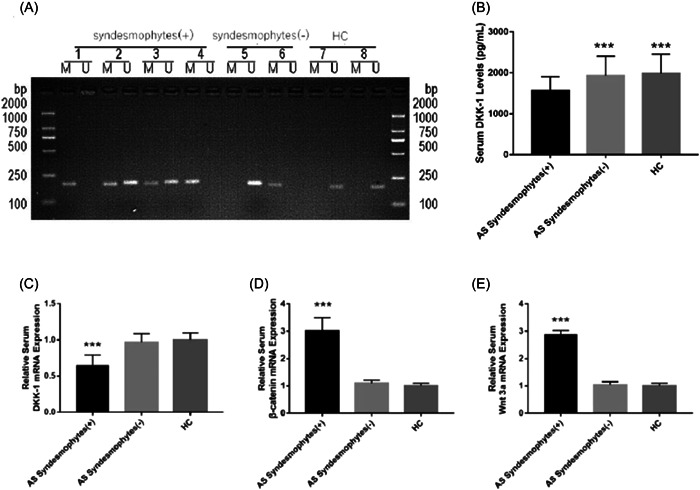
(A) The methylation status of serum dickkopf‐associated protein 1 (DKK‐1) gene promoters among ankylosing spondylitis (AS) with syndesmophytes group (*n* = 36), AS with no syndesmophytes group (*n* = 40) and control group (*n* = 42). (B) Comparison of serum DKK‐1 expressions among AS with syndesmophytes group (*n* = 36), AS with no syndesmophytes (*n* = 40) and healthy controls (*n* = 42). (C) Comparison of serum DKK‐1 messenger RNA (mRNA) levels among AS with syndesmophytes group (*n* = 36), AS with no syndesmophytes group (*n* = 40) and control group (*n* = 42). (D) Comparison of serum β‐catenin mRNA levels among AS with syndesmophytes group (*n* = 36), AS with no syndesmophytes group (*n* = 40) and control group (*n* = 42). (E) Comparison of serum Wnt3a mRNA levels among AS with syndesmophytes group (*n* = 36), AS with no syndesmophytes group (*n* = 40) and control group (*n* = 42). ***p < .001 versus AS with syndesmophytes group.

Next, the serum levels of methylated DKK‐1 were detected using MSP among 36, 40, and 42 in AS with syndesmophytes group, AS with no syndesmophytes group and healthy control group, respectively. Results showed that the serum methylated DKK‐1 was observed in 26 patients in AS + syndesmophytes group (72.1%) compared with 10 patients in AS with no syndesmophytes group (23.8%) and 5 control group (11.9%) (Figure 2). In contrast to Group 2 (*χ* ^2^ = 6.11, *p* = .014) and control group (*χ* ^2^ = 12.87, *p* < .001), the serum DKK‐1 GPM rate in AS + syndesmophytes group was higher with statistical significance (Figure 2). We also detected serum DKK‐1 expressions among three groups. We found serum DKK‐1 levels were significantly decreased in AS with syndesmophytes group in comparison with AS with no syndesmophytes and healthy controls (1555.0 ± 348.5 vs. 1925.5 ± 482.3 vs. 1979.3 ± 475.5 pg/mL, *p* < .001) (Figure 2B). Furthermore, serum DKK‐1 mRNA, β‐catenin mRNA, and Wnt3a mRNA levels in AS patients and healthy controls were also investigated. We found serum levels of DKK‐1 protein and mRNA in AS with syndesmophytes group were markedly decreased (Figure 2C), while β‐catenin mRNA (Figure 2D) and Wnt3a mRNA (Figure 2E) expressions were significantly increased than AS with no syndesmophyte group and healthy control group.

### 3.3 The serum DKK‐1 methylation rate

**Figure 3:**
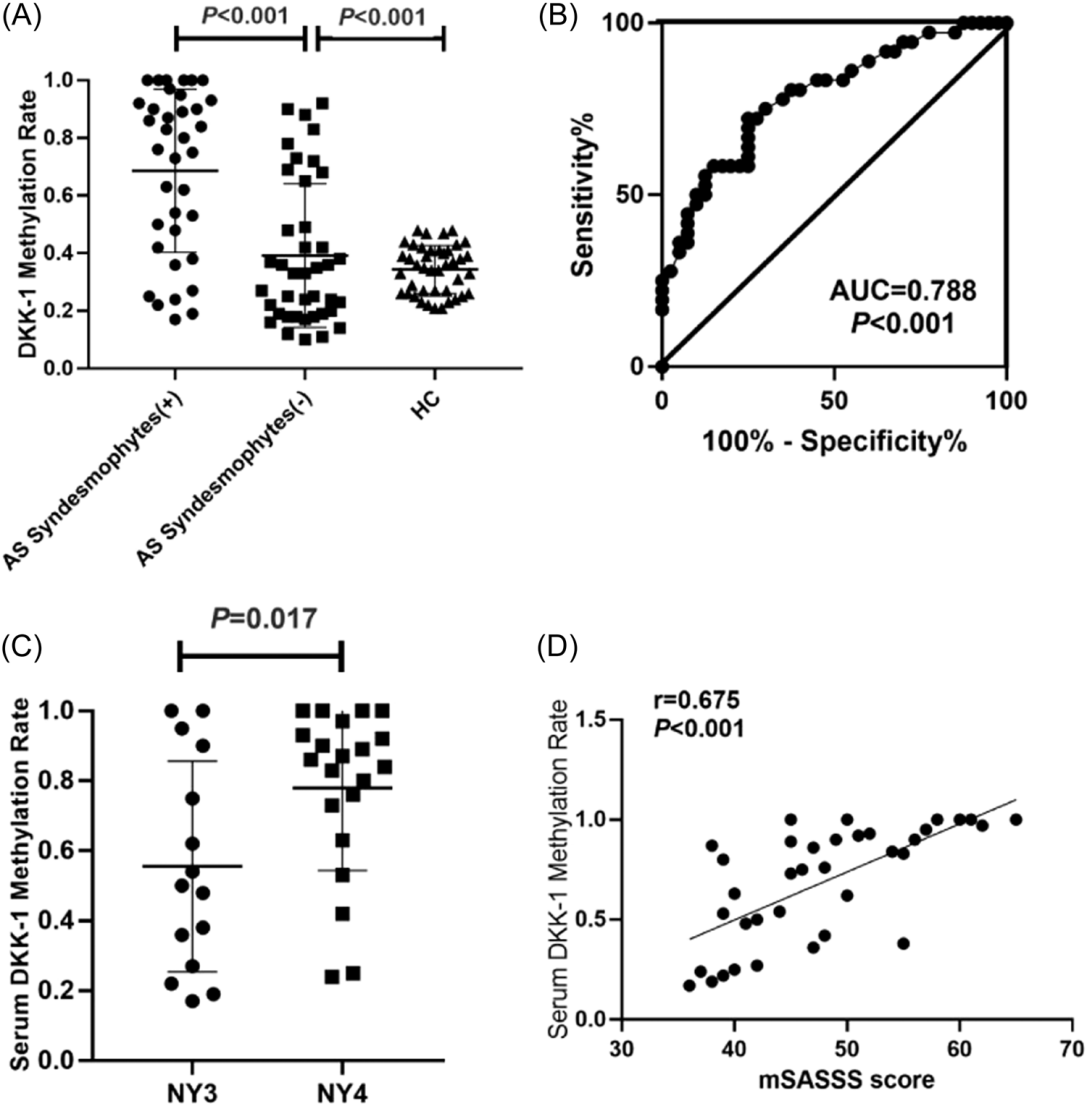
Serum dickkopf‐associated protein 1 (DKK‐1) methylation rate in ankylosing spondylitis (AS) patients with syndesmophytes (AS + syndesmophytes group, *n* = 36), AS patients without syndesmophytes (AS group, *n* = 40), as well as healthy individuals (control group, *n* = 42). (A) Distribution of serum DKK‐1 methylation rate in AS + syndesmophytes group, AS group, as well as the control group. (B) Receiver operating characteristic curve of DKK‐1 methylation rate with regard to syndesmophytes (−) (*n* = 15) versus syndesmophytes (+) (*n* = 21). (C) Comparison of serum DKK‐1 methylation rate in AS patients at Grade 3 versus Grade 4 based on modified New York criteria. (D) Correlation of methylation rate of DKK‐1 with modified Stoke Ankylosing Spondylitis Spinal Score (mSASSS).

Based on real‐time fluorescence MSP, the serum DKK‐1 methylation rate was determined and results showed that in contrast to AS with the no syndesmophytes group and control group, DKK‐1 gene methylation rate was significantly higher in AS + syndesmophytes group (Figure 3A). An ROC curve was drawn and demonstrated a good diagnostic performance of serum DKK‐1 methylation rate with regard to the AS abnormal bone forming (area under cureve = 0.788, *p* < .001).

### 3.4 Correlation of the serum DKK‐1 GPM rate with radiographic progression

We further analyzed parameters of DKK‐1 methylation rate with radiographic progression in AS patients. On one hand, the serum DKK‐1 GPM rates were higher in AS patients at Grade 4 compared with those at Grade 3 based on mNYC (*p* = .017) with statistical significance (Figure 3C). On the other hand, the serum DKK‐1 GPM rates were positively correlated with mSASSS (*r* = .675, *p* < .001) (Figure 3D), indicating a higher serum DKK‐1 GPM rate among AS patients with an advanced radiographic progression.

## 4 DISCUSSION

Epigenetic modification is an important mechanism for regulating gene expression and studies have demonstrated that DNAm exerts an increasingly significant impact on the occurrence/development of AS.^29^, ^30^, ^31^ However, there is no study illustrating the possibility of DKK‐1 methylation in AS. This study is the first time to detect the expression of DKK‐1 and methylation status in ossified hip capsule synovium obtained from patients with AS. The DKK‐1 protein expression was found to be significantly lower and the DKK‐1 methylation level was significantly increased compared with the control group. These findings suggested that DKK‐1 hypermethylation may correlate with the pathological bone formation of AS, which exerts a significant impact on the pathogenesis, diagnosis, and treatment of AS.

In this paper, DKK‐1 was downregulated in the ossified hip capsule synovium of AS patients, which was consistent with the findings of our previous study^11^ and Daoussis et al.,^32^ suggesting that the downregulation of DKK‐1 was involved in the progress of pathological ossification of AS. In addition, we found that DKK‐1 was hypermethylated, suggesting that the downregulation of DKK‐1 may be partially attributed to hypermethylation.

So far, DNAm has made progress in the diagnosis/treatment of various diseases, represented by the methylation detection kit for colorectal cancer—septin9^33^ and sdc2^34^ methylation detection kit. Meanwhile, 5‐azacytidine, a demethylated drug, has already been used to treat leukemia and MDS.^35^ Recently, it has also been invested in the basic research on the intervention of lung cancer,^36^ cervical cancer,^37^ RA,^38^ and chronic obstructive pulmonary disease.^39^ The potential role of DKK‐1 methylation with regard to AS pathological bone formation was investigated. One previous study showed that during the normal osteoblastic differentiation of bone marrow‐derived mesenchymal stem cells, the promoter of the DKK‐1 gene remains unmethylated or hypo‐methylated.^40^ However, in our study, we found DKK‐1 become methylated under pathological conditions of AS abnormal bone formation.

Some limitations existed in our study. First, the sample size of this study was small, thus a larger sample size is of necessity in the future. Second, no intervention test was conducted to verify whether the demethylation of 5‐azacytidine could reverse the downregulation of DKK‐1 and the heterotopic ossification. Third, due to ethical restrictions, it is impossible to collect samples from patients with short or mild to moderate disease course or different disease stages of the same patient to further analyze the dynamic changes of DKK‐1 during the progression of AS disease. In the future, we will longitudinally observe the changes of DKK‐1 as well as DKK‐1 methylation in the progression of AS, so as to provide new disease markers and diagnosis and treatment methods for AS. Lastly, we did not consider the potential effect of the drugs on the DKK‐1 methylation rate.

In conclusion, our study showed that DKK‐1 hypermethylation may contribute to the pathological bone formation of AS, providing novel strategies for the intervention of abnormal bone formation in AS patients. Investigation of different drugs targeting DKK‐1 methylation to inhibit abnormal bone formation in AS deserves further study in the future.

## AUTHOR CONTRIBUTIONS

**Yu‐Cong Zou**: Review and editing (equal). **Zi‐Rui Luo**: Conceptualization (lead); writing—original draft (lead); formal analysis (lead); writing—review and editing (equal). **Yi‐Feng Lan**: Software (lead); writing—review and editing (equal). **Jia‐Yu Yao**: Software (lead); writing—review and editing (equal). **Zhi‐Hui Yu**: Methodology (lead); writing—review and editing (equal). **Zhi‐Jun Wang**: Methodology (lead); writing—review and editing (equal). **Li‐Cheng Shao**: Conceptualization (supporting); writing—original draft (supporting); writing—review and editing (equal).

## ETHICS STATEMENT

The present study was approved by the 5th People's Hospital of Foshan City. All the patients and investigators consented to participate in this study.

## Data Availability

The datasets used and/or analyzed during the current study are available from the corresponding author on a reasonable request.
